# Effectiveness of IT-supported patient recruitment: study protocol for an interrupted time series study at ten German university hospitals

**DOI:** 10.1186/s13063-024-07918-z

**Published:** 2024-02-16

**Authors:** Martin Boeker, Daniela Zöller, Romina Blasini, Philipp Macho, Sven Helfer, Max Behrens, Hans-Ulrich Prokosch, Christian Gulden

**Affiliations:** 1 Institute of Medical Biometry and Statistics, Medical Faculty and Medical Center, University of Freiburg, Freiburg im Breisgau, Germany; 2 Chair of Medical Informatics, Institute of Artificial Intelligence and Informatics in Medicine, Klinikum rechts der Isar, School of Medicine and Health, Technical University of Munich, Munich, Germany; 3 Institute of Medical Informatics, Justus-Liebig-University Gießen, Gießen, Germany; 4 Institute of Medical Biostatistics, Epidemiology and Informatics (IMBEI), Mainz University Medical Center, Mainz, Germany; 5 Department of Pediatrics, Faculty of Medicine and University Hospital Carl Gustav Carus, Technische Universität Dresden, Dresden, Germany; 6 Chair of Medical Informatics, Department of Medical Informatics, Biometrics and Epidemiology, Friedrich-Alexander Universität Erlangen-Nürnberg, Erlangen, Germany

**Keywords:** Clinical trial, Clinical trial recruitment support, Patient recruitment, Hospital information system

## Abstract

**Background:**

As part of the German Medical Informatics Initiative, the MIRACUM project establishes data integration centers across ten German university hospitals. The embedded MIRACUM Use Case “Alerting in Care - IT Support for Patient Recruitment”, aims to support the recruitment into clinical trials by automatically querying the repositories for patients satisfying eligibility criteria and presenting them as screening candidates. The objective of this study is to investigate whether the developed recruitment tool has a positive effect on study recruitment within a multi-center environment by increasing the number of participants. Its secondary objective is the measurement of organizational burden and user satisfaction of the provided IT solution.

**Methods:**

The study uses an Interrupted Time Series Design with a duration of 15 months. All trials start in the control phase of randomized length with regular recruitment and change to the intervention phase with additional IT support. The intervention consists of the application of a recruitment-support system which uses patient data collected in general care for screening according to specific criteria. The inclusion and exclusion criteria of all selected trials are translated into a machine-readable format using the OHDSI ATLAS tool. All patient data from the data integration centers is regularly checked against these criteria. The primary outcome is the number of participants recruited per trial and week standardized by the targeted number of participants per week and the expected recruitment duration of the specific trial. Secondary outcomes are usability, usefulness, and efficacy of the recruitment support. Sample size calculation based on simple parallel group assumption can demonstrate an effect size of *d*=0.57 on a significance level of 5% and a power of 80% with a total number of 100 trials (10 per site). Data describing the included trials and the recruitment process is collected at each site. The primary analysis will be conducted using linear mixed models with the actual recruitment number per week and trial standardized by the expected recruitment number per week and trial as the dependent variable.

**Discussion:**

The application of an IT-supported recruitment solution developed in the MIRACUM consortium leads to an increased number of recruited participants in studies at German university hospitals. It supports employees engaged in the recruitment of trial participants and is easy to integrate in their daily work.

**Supplementary Information:**

The online version contains supplementary material available at 10.1186/s13063-024-07918-z.

## Background

Clinical trials are the most important method for gaining new insights into the connection between medical treatments, environmental influences, and other factors on people’s health. Furthermore, only randomized controlled trials (RCTs) can provide conclusive evidence for causal effectiveness of new drugs and medical procedures on health. As the main link in the chain of evidence, RCTs are crucial for translating scientific results from basic research to clinical application [1, 2].

The success of clinical trials depends on the enrollment of a sufficiently large patient population. Failure to do so will result in reduced statistical power, ineffective use of human resources, and costly extensions to the trial duration [3, 4]. However, achieving the planned recruitment numbers remains one of the biggest challenges when conducting clinical trials [5–7]. As a consequence, sites in many countries have diminishing opportunities to participate in international studies and contribute to scientific progress. Further, many patients lose the chance to benefit from novel therapies that might have positive effects on their prognosis and quality of life [6, 8].

As an alternative to manual identification of eligible persons, technical solutions using data from electronic health records (EHR) have been developed to improve the inclusion of patients in clinical trials [9–11]. Initially aimed at supporting trial planning, those IT solutions informed how many patients with defined characteristics are available at a trial site (feasibility) [12]. Recently, there have been many approaches that help screening for eligible patients in the hospital information system (HIS) and directly suggest their inclusion into running trials [10, 11, 13, 14]. For some of these solutions, improvements in participant recruitment have been shown [15]. However, existing solutions often had either severe technical or methodological limitations or were not evaluated across multiple clinical sites.

The goal of the MIRACUM project [16], which is part of the German Medical Informatics Initiative [17], is to establish data integration centers across ten German university hospitals. The data integration centers shall transform the large and heterogenous amount of patient data into harmonized research repositories at each site. To demonstrate their effectiveness, three use cases in different clinical and application contexts were defined. Use Case 1 (UC1, Alerting in Care - IT Support for Patient Recruitment), aims to support the recruitment into clinical trials by automatically querying the repositories for patients satisfying the eligibility criteria and presenting them as screening candidates. To accomplish this, we developed software which leverages the OMOP Common Data Model (CDM) [18] and HL7 FHIR to provide an interoperable and open technical infrastructure for supporting the patient recruitment process [19].

The objective of this study is to investigate whether the developed recruitment tool has a positive effect on trial recruitment within a multi-center environment by increasing the number of participants. The secondary objective is the measurement of organizational burden and user satisfaction of the provided IT solution.

In the following, the presented evaluation is denominated as “study” and the clinical studies forming our target population as “trials”.

### Objectives

#### Hypotheses

The application of an IT-supported recruitment solution developed in the MIRACUM Use Case 1 (UC1) leads to an increased number of recruited participants in studies at German university hospitals. It supports employees engaged in recruitment of trial participants and is easy to integrate in their daily work.

#### Primary objective

To investigate whether the application of an IT-supported recruitment solution developed within the MIRACUM consortium is effective in increasing the number of recruited trial participants in studies at German university hospitals.

#### Secondary objective

To survey the subjective experience of the recruitment staff with the solution on (a) usability, (b) user satisfaction, and (c) efficacy, i.e., the support of the recruitment and inclusion processes.

## Methods

This study protocol has been developed adhering to the SPIRIT 2013 statement: Defining standard protocol items for clinical trials [20]. The completed SPIRIT 2013 Checklist is available as Supplementary File 1.

### Study design

The study uses an Interrupted Time Series (ITS) Design [21, 22] with a duration of 15 months. All trials start in the control phase of randomized length with regular recruitment and change to the intervention phase with additional IT support, ensuring a minimum of one month in each phase and a minimum trial observation of 6 months. At least one month prior to the inclusion of *a* specific trial into the study, the length of the control phase for this trial is randomized centrally across all 10 sites. A block randomization was used to randomize the time of switching to the new scheme in the percentage of the planned enrollment time into the study. We used R version 4.1.2 and the package blockrand version 1.5 with the seed 9384902. We allowed for values between 0.15 and 0.85 with 0.05 steps, rounding the resulting time of switching to the first or 15th of a month. We generated 5 blocks with a size of 30 resulting in 150 ids. When a study was enrolled at one of the sites, the site contacted the independent person at Freiburg, who took the next free id from the randomization list and relayed the time of switching for the specific study. The persons responsible for enrollment had no access to the randomization list.

Due to the embedding of the intervention into and its overlap with the recruitment processes, it is practically not feasible to completely blind the study. Nevertheless, the randomized time point to switch to the intervention will only be disclosed to the sites 2 months before and only subsequently to the medical staff. The effectiveness will be measured by the number of recruited participants per trial and week standardized by the targeted number of participants to be recruited into the specific trial. This will be analyzed by means of a linear mixed model. In addition, user interviews will be conducted prior to the start of the intervention and at the end of the study. 

### Settings

**Fig. 1:**
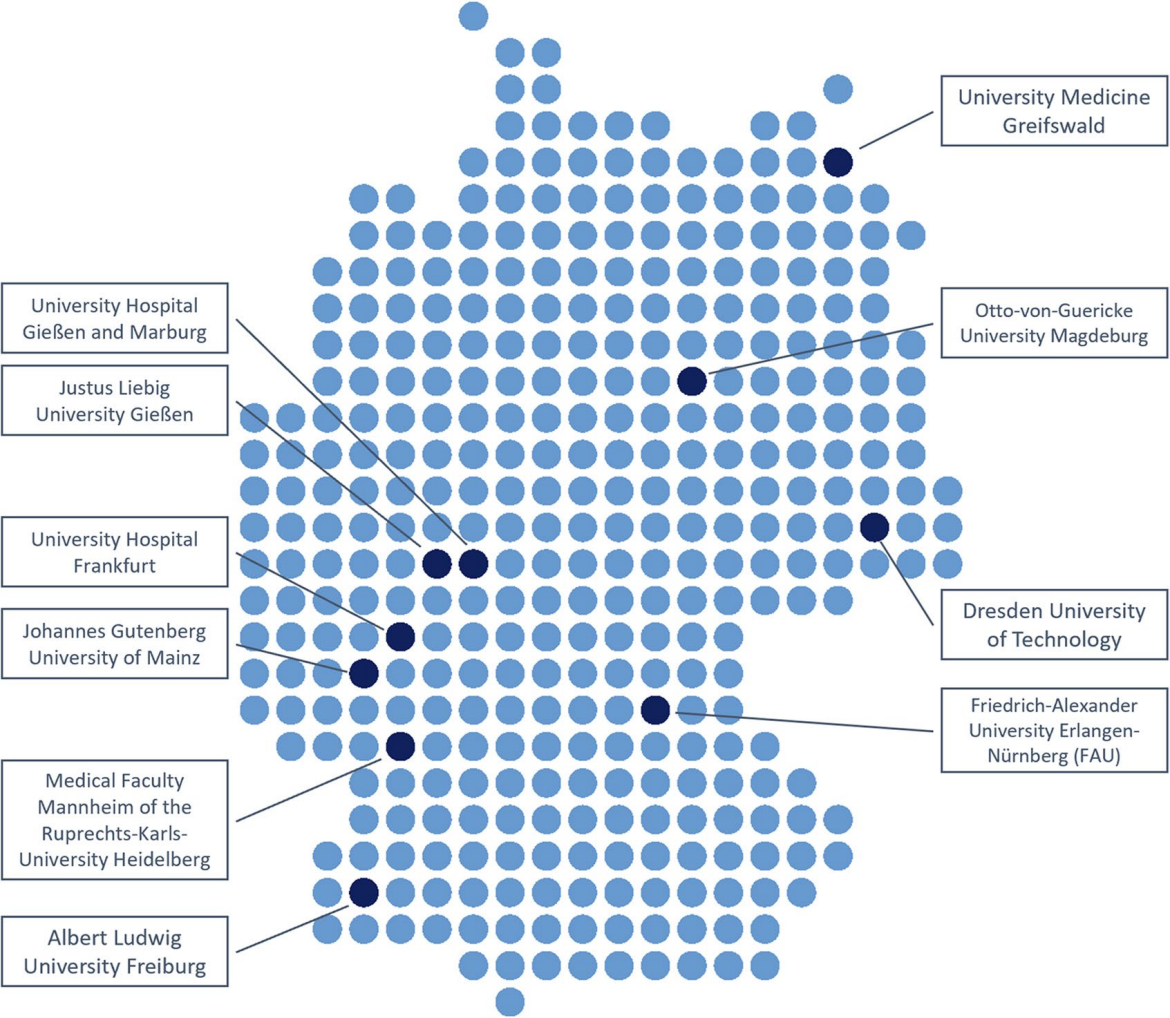
Location of the participating university hospitals across Germany

The study will be conducted at all university hospitals of the MIRACUM consortium partners within the BMBF Medical Informatics Initiative in the years 2021/2022 (Fig. 1):

- Dresden University of Technology
- Friedrich-Alexander University of Erlangen-Nuremberg
- University Hospital Frankfurt
- Albert Ludwig University Freiburg
- Justus Liebig University Giessen
- University Medicine Greifswald
- Otto-von-Guericke University Magdeburg
- Johannes Gutenberg University of Mainz
- Medical Faculty Mannheim of the Ruprechts-Karls-University Heidelberg
- University Hospital Gießen and Marburg

### Participants

#### Eligibility criteria

Trials fulfilling the following criteria are included into the study:

- The specified inclusion and exclusion criteria can be formalized in a machine-processable way
- The recruitment phase of the trial is overlapping at least 6 months with the observation period of the study. Recruitment of patients into the trial is expected during the observation period.
- An individual trial can take part at one site or multiple sites. Usually, it takes part only at one site and is then included in the study as a single trial at the corresponding site; if *n* sites participate in a trial the trial is included *n* times at the corresponding sites.
- Prospective evaluation

Trials meeting the following criteria are excluded:

- Animal experimental studies
- Biomaterial studies
- Trials recruiting patients with psychiatric diagnoses
- Pilot or feasibility trials

### Intervention

**Fig. 2:**
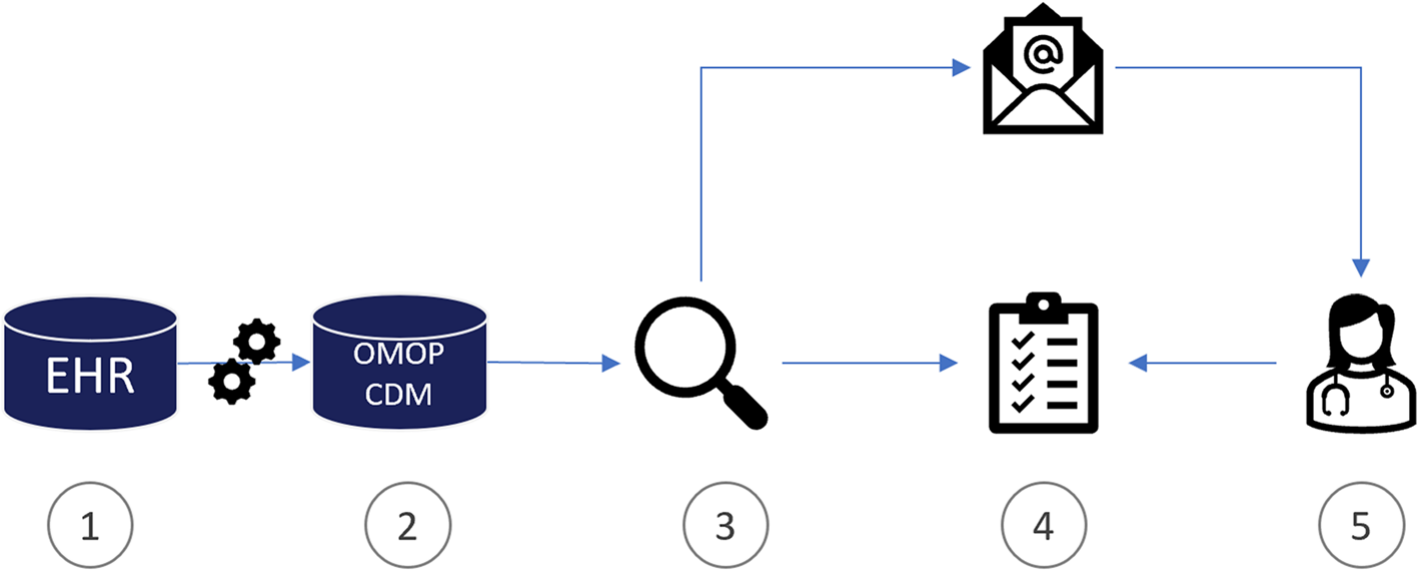
The basic flow of data from the electronic health records (EHRs) to the research repositories to the creation of patient recommendations in the screening list. Treatment data is recorded in the EHR as part of routine care (1), this data is regularly transformed to the OMOP CDM via ETL jobs (2). A query module (3) continuously scans the research repository for patients matching the defined trial eligibility criteria. If potential candidates are found, they are put on a web-based screening list (4), and a notification is sent via email. This notification is received by relevant practitioners or trial personnel (5) and the screening list can be accessed to further manually screen the suggested patients

The intervention consists of the application of a complex recruitment-support system, which should be utilized alongside the standard recruitment procedure. Patient data, which is already collected in the context of general care, will be used for screening according to trial-specific criteria. The de-identified patient data, located in the data integration centers of the sites, are continuously compared against inclusion and exclusion criteria of clinical trials. This requires first translating the necessary eligibility criteria of all selected trials into a machine-readable format. The recruitment support system uses the OMOP common data model [18] to store patient data which includes OHDSI ATLAS as a tool for defining patient cohorts. ATLAS is a user interface where researchers can graphically define a cohort using a set of logical expressions that are internally converted to SQL to be executed against the database. This tool is used to identify and translate the eligibility criteria collaboratively with the trial personnel [23, 24]. During the intervention phase, all patient data is regularly checked against the defined cohorts. When eligible patients are detected, the staff is informed about the proposals by email, and the de-identified data is presented on a web-based screening list. The dataflow is shown in Fig. 2.

Local recruitment staff decides over inclusion of individual patients based on the recruitment proposals on the screening list. Patient status, from recruitment proposal to inclusion, is documented in the screening list. Because it is based on pseudonymized research data, by default, this list displays only a pseudonymized medical record number, the year of birth, the gender, and the last known organizational unit within the hospital. This data can be used to either search the hospital information system with its applicable access controls, or the study personnel has to contact the attending physician based on the last known location of the patient. After consenting to disclose trial and personal data, the staff can then inform the patient about a possible participation in the trial. Some sites allow displaying the re-identified medical record number to make it easier to identify the patient. In these cases, re-identification works by technically integrating the screening list with the pseudonymization tooling established at the sites.

In the control phase, trial participants are recruited according to the respective standard procedure.

Termination criteria are not defined, since the interventional IT solution is used parallel to the conventional solution.

To increase the adherence of the recruiting staff, training courses are conducted and regular visits take place. Problems with adherence are logged.

### Outcomes

#### Primary outcome

Absolute and relative difference in the recruitment numbers of the observed studies before and after the intervention, in relation to the actual recruitment time. The primary outcome is the number of participants recruited per trial and week standardized by the targeted number of participants per week and the expected recruitment duration of the specific trial (actual recruitment number in week *i*/expected recruitment number per week, where expected recruitment number per week = target number of cases/duration of the trial in weeks and *i*=1, ..., observation period in week *i*, *j*=trial 1, ...,5, *k*=site 1, ...,10).

To account for site-specific and seasonal effects, a mixed model will be used.

#### Secondary outcomes

The absolute numbers of

- Patients identified
- Patients incorrectly identified
- Patients recruited

per time-period, site, and trial, and contrasted for control and intervention phases.

 Determination of the usability, usefulness, and efficacy of the recruitment support.

#### Time schedule

*Recruitment*: Trials are included starting on June 1, 2021.

*Randomization*: At least one month prior to the inclusion of *a specific* trial into the study, the length of the control phase for this trial is randomized centrally across all 10 sites.

#### Sample size calculation

Sample size calculation is based on the assumption of a simple parallel group design. On a significance level of 5% and a power of 80%, an effect size of *d*=0.57 can be demonstrated with a two-sided *t*-test in a balanced design with a total number of 100 trials (10 per site). Even if this effect can be classified as strong, the dynamic Interrupted Time Series design offers more statistical power than a two-group t-test, especially when the number of data points is relatively large and the effect size is not small, as is expected in our case. This added power in the ITS design is mainly due to its methodology as it incorporates the temporal ordering of observations, which allows it to account for time-related effects. Thus, it can potentially provide a more accurate estimate of the intervention effect, especially when considering various variability factors (time, location). In addition, we aim to include as many trials as possible, potentially exceeding the total number of 100.

To assess the feasibility of this number, we searched ClinicalTrials.gov for trials registered and actively recruiting that began recruitment on 01-01-2021 or earlier and whose primary completion date is 01-01-2023 or later. Table 1 shows the results per site.

**Table 1:** Number of recruiting trials at the participating sites with a start date on or before 01-01-2021 and primary completion on or after 01-01-2023 with the location filter set to the city name of the site

Site	Number of trials on ClinicalTrials.gov satisfying criteria for feasibility estimation
University Hospital Dresden	185
University Hospital Erlangen	214
University Hospital Frankfurt	189
University Hospital Freiburg	103
University Hospital Gießen	73
University Hospital Greifswald	29
University Hospital Magdeburg	58
University Hospital Mainz	116
University Hospital Mannheim	75
University Hospital Marburg	66

### Recruitment

To identify large and viable trials for the study, central and site-local trial registries are searched and trial personnel are contacted directly to contribute additional trials. To increase the sample size of the study, any eligible trial can be dynamically included in the study and allocated to a randomized time slot, generated prior to the start of the trial.

### Generation and implementation of allocation

Prior to the inclusion of *a specific* trial into the study, the length of the control phase for this trial is randomized centrally across all 10 sites. The overall observation period of the trials varies between 6 and 15 months. With a pseudo-random generator from the R-statistical package, the length of the control phase is randomly assigned to a value between 15% and 85% of the total observation period, which ensures that for short (6 months) trials control or intervention phases have at least a duration of one month. Respectively, a trial with an observation time of 15 months has control or intervention phases with a minimum length of 2.5 months. For each trial, an exact date for the start of the intervention phase is calculated from the inclusion date of the trial and the duration of the control phase.

### Blinding

Due to the embedding of the intervention into and its overlap with the recruitment processes, it is practically not possible to completely blind the study. Nevertheless, the randomized time point to switch to the intervention will only be disclosed to the site’s medical staff 1 month before. The randomization list is generated prior to the start of the evaluation study by an independent statistician and kept secret.

### Data collection

The following data is collected for each trial:

Trial specific parameters

1. Participating sites
2. Registration information: unique trial identifier such as the ClinicalTrials.gov NCT number, the DRKS number, or the Universal Trial Number (UTN)
3. Start of recruitment
4. Planned and actual end of recruitment
5. Expected duration of recruitment
6. Targeted participant count
7. Expected number of participants at each site
8. Number of participants already recruited at the beginning of the study
9. Trial type
10. Inclusion criteria
11. Exclusion criteria
12. Persons (or groups) involved in recruitment
13. Number of patients who were admitted to the site on a monthly basis as in- or outpatients

During the intervention:

1. Number of screenings conducted
2. Number of recruited participants per week
3. Number of recruitment proposals per week (automatically recorded by the IT-solution)
4. Number of recruited participants not suggested by the system per week (i.e., only through standard recruitment procedures)

After the intervention:

Short interviews and surveys on the usability of the system are conducted with one user for each trial. To quantify the user experience and usability of the tool, the System Usability Scale (SUS) [25] and the User Experience Questionnaire - short (UEQ-S) [26] will be used.

Per recruitment proposal:

The following data is collected for each recruitment proposal. A suitable feedback mechanism for reporting trial inclusions must be established individually at the centers.

1. Organizational unit of the site where the inclusion took place (automatically recorded by the IT system)
2. Status as indicated in the recruitment list.
  - “Recruitment Proposal”
  - “Under consideration”
  - “Does not meet eligibility criteria”
  - “Is Participating in trial”
  - “Not willing to participate”
3. Outpatient or inpatient status at the time of inclusion

The patient inclusion data is primarily collected via the trial’s existing electronic documentation and transferred to custom data entry masks. To ensure the completeness of data collection, the documentarians from MIRACUM are in close exchange with the corresponding staff in the departments and trial personnel.

### Data management

REDCap is used as a data acquisition tool.

Data on individual patients (the minimal amount of data needed to present patients as screening recommendations) are stored pseudonymized, in accordance with the local data protection regulations.

### Statistical methods

The characteristics of the included studies will be presented in tabular form. All variables will be summarized using appropriate summary statistics. The mean, standard deviation, and the quantiles will be used for continuous variables and absolute and relative frequencies for categorical variables. If applicable, the variables will be visualized using boxplots and histograms, potentially over time.

The primary analysis will be conducted using linear mixed models with the actual recruitment number per week and trial standardized by the expected recruitment number per week and trial as the dependent variable. The choice is driven by the model’s ability to account both for fixed effects and random effects. This flexibility of this model is particularly suited for capturing the hierarchical structure in our data and aligns with the nature of the ITS design, where repeated measures over time and within-trial correlations are essential. A positive and statistically significant fixed effect of the binary variable “intervention allocation per week” will be interpreted as the measure for effectiveness. The corresponding effect will be evaluated for significance on the 5% level using the likelihood-ratio test. The model will be adjusted for trial, site, season, and time via random effects. Primarily, compound symmetry is considered as a working covariance matrix (constant correlation between time points), while autoregressive and unstructured covariance matrices are used as sensitive analysis. Additional sensitive analysis involves larger time frames, i.e., per month instead of per week, other distributions and link functions, i.e., generalized models, and classical statistics via mean values. Given the conservative sample size calculation based on a *t*-test, the linear mixed model is backed with adequate statistical power given the expected heterogeneous nature of the data.

To uncover further correlations and generate hypotheses, explorative analysis is carried out, including specific evaluation of site and trial characteristics.

No deviations from the protocol or missing data are expected. Trials are otherwise excluded from the primary analysis and their characteristics are compared to the other trials to identify potential problems for generalizability of the results.

### Data monitoring

A Data Monitoring Committee (DMC) is not used, as there can be no undesired effects of the intervention, since the IT-supported intervention tool is only used in addition to the standard method during trial recruitment.

A premature termination of the study is not planned.

### Risks and measures for error management

Since the intervention does not directly affect the patient, but is only used in addition to the standard recruitment procedure, no direct risks to patients are expected.

Patients who might be incorrectly proposed for trial inclusion will be recognized as such by the physician or trial assistant review and not included. This will happen as soon as possible with no additional burden for the patient.

A low risk may only result from the additional use of resources (personnel and time) when interacting with the recruitment support tool and the need for additional documentation.

Rather, we expect primarily positive effects for patients who otherwise would not have been identified as trial patients and could be proposed for inclusion due to the use of the application.

### Protocol amendments

Possible protocol extensions are formally added to the study protocol.

### Information and consent

Within the framework of the study, pseudonymized data are processed within the data protection laws of the individual sites and in the context of supporting medical care and research. The evaluation itself does not involve data from trial participants.

### Data protection and confidentiality

GDPR-conformaning privacy protection and data usage are guaranteed by an overarching central research program (BMBF Medical Informatics Initiative) and an overall consortial (MIRACUM) data protection concept. Data protection concepts are approved by the national conference of data protection officers and the data protection officers in the participating sites.

The complex process of identifying patients based on pseudonymized data using either the hospital information system or by contacting the treating physicians allows the system to be conformant with data protection regulations by relying on the access control mechanisms of the hospital information systems and established cooperation agreements between organizational units.

The study only collects the data listed under “Data collection.” The data is collected in a secure environment at each site.

### Declaration of competing interests

The authors declare no competing interests.

### Access to data

The original data remain under the sovereignty of the respective locations. The data will be further processed according to the vote of the data protection, the ethics committee, and the Use & Access Committee. The aggregated results will be published and used jointly in the MIRACUM Consortium under the sovereignty of the MIRACUM Steering Board.

For statistical analysis, the collected data of each site is processed at one dedicated site within the consortium.

### Dissemination

The results are disseminated through publications at conferences and in specialist journals. The software components used in the intervention will be made freely available as open source, unless subject to further restrictions.

## Discussion

The evaluated IT solution is deployed on the common technical infrastructure of all partnering sites, leveraging international standards for interoperability. However, it is also deployed in a diverse setting of established organizational standards, tools, and processes. Therefore, user engagement is foundational to the success of the system. Ultimately, the implemented solution should benefit both trial personnel by reducing the manual effort required to identify eligible patients across large amounts of data and the patients themselves by providing them with access to promising treatment options within the trial.

### Supplementary Information

**Additional file 1.**

## Data Availability

The developed software solution is publicly available under an open source license for download and usage at https://github.com/miracum/recruit.

## Abbreviations

EHR: Electronic health record
CDM: Common data model
ETL: Extract transform load
RCT: Randomized controlled trial
HL7: Health level 7
FHIR: Fast healthcare interoperability resources
OMOP: Observational medical outcomes partnership
HIS: Hospital information system
MIRACUM: Medical informatics in research and medicine
OHDSI: Observational health data sciences and informatics
DMC: Data Monitoring Committee
SUS: System usability scale
UEQ-S: User Experience Questionnaire - short
ITS: Interrupted time series
